# Global and national burden of peripheral arterial disease from 1990 to 2021: a systematic analysis of global burden of disease study 2021

**DOI:** 10.3389/fcvm.2025.1603810

**Published:** 2025-10-27

**Authors:** Wei Liu, Chi Cui

**Affiliations:** Center of Vascular and Interventional Surgery, Department of General Surgery, The Third People’s Hospital of Chengdu, Affiliated Hospital of Southwest Jiaotong University & The Second Affiliated Hospital of Chengdu, Chongqing Medical University, Chengdu, China

**Keywords:** age-standardized rate, estimated annual percentage change, global burden of disease, peripheral arterial disease, epidemiology

## Abstract

**Background:**

Peripheral arterial disease (PAD) poses a significant global health challenge. Comprehensive global assessments of the disease burden of PAD remain limited. The study utilizes the Global Burden of Disease (GBD) framework to analyze global trends in PAD systematically from 1990 to 2021. Furthermore, future trends through to 2035 are projected.

**Methods:**

Data of PAD from the GBD Study 2021 was employed, covering incidence of PAD, mortality rates, disability-adjusted life years (DALYs), age-standardized incidence rate (ASIR), age-standardized mortality rate (ASMR), and age-standardized disability-adjusted life years (ASDALYs) across 204 countries and regions from 1990 to 2021. Trend projection up to the year 2035 was performed with the Bayesian age-period-cohort model.

**Results:**

Globally, the ASIR, ASMR and ASDALYs of PAD exhibited a downward trend in the years studied. The age analysis showed that the proportion of the population aged 70 and over with PAD exceeded 90%, whether in terms of ASIR, ASMR, or ASDALYs. These metrics showed an upward trend for people with PAD under 70 years old. The primary risk factor for PAD was smoking. Our projections show that, from 2022 to 2035, the ASIR (115.2–110.5), ASMR (0.8–0.7) and ASDALYs (18.5–16.5) of PAD will all follow a downward trend.

**Conclusions:**

Overall, the global disease burden of PAD shows a downward trend. However, it exhibits an upward trend in regions with low and middle-low socio-demographic index. The cessation of smoking should be the primary public health measure to continue this trend.

## Introduction

Peripheral arterial disease (PAD) is an atherosclerotic cardiovascular disease (ASCVD) affecting over 236 million people worldwide. PAD is the third leading cause of ASCVD after ischemic heart disease and cerebrovascular disease (1–3). Along with the added burden of atherosclerosis, PAD patients are at an increased risk for major adverse cardiovascular events, and of all-cause mortality (2, 4, 5). The 2019 Global Burden of Disease Study (GBD) claims that 1.5 million disability-adjusted life years (DALYs) are caused by PAD worldwide (6), making this condition a major problem for public health. Currently, the data from the GBD 2021 has been updated. It remains unclear whether there have been changes in the global disease burden of PAD. Therefore, we conducted this study.

The main objectives of this study were to evaluate the incidence of PAD, mortality, DALYs, and risk factors, and to predict the trend of DALYs up to 2035 using the updated GBD 2021 database. We conduct a further comparison of the incidence rates, mortality rates, and DALYs of PAD across different countries and regions. This information can offer insights from multiple perspectives. Specifically, first, the GBD database encompasses over 200 countries and regions globally. By comparing the relevant data of different countries, we can clearly observe the global distribution of PAD. Second, comparing mortality and incidence rates can reveal the differences in the development trends of PAD, which is conducive to analyzing the achievements and deficiencies in PAD prevention and control in different countries or regions. Third, the risk factors for PAD vary across different countries and regions. Through comparison, the primary risk factors in each country and region can be identified. Finally, by comparing the data from different countries and regions, it can provide references for national governments in formulating health policies, contribute to the development of more effective intervention measures and resource allocation plans, clarify the priorities and challenges in the global health field, and promote cooperation and exchanges among countries to jointly address global health challenges.

## Methods

### Data source and data collection

This retrospective analysis of PAD incidence, mortality and DALYs made use of data collected for the GBD study. Estimates for these factors were extracted from online data repositories; data from 1990 to 2021 were analyzed. These methods have been published previously (7). The incidence data in the GBD database are not directly collected but estimated through the integration of multiple data sources and the application of specific models and methods. The data sources of the GBD database include death registration, disease surveillance, population censuses, household surveys, clinical records, health service data, etc., and also involve innovative data sources such as satellite remote sensing and social media. The research team uses Bayesian meta - regression tools like DisMod - MR to conduct comprehensive analysis and modeling of these data, thereby estimating the incidence rates of different diseases in different regions, among different age groups, genders, and at different time points. The GBD database indeed provides incidence and prevalence data at different time points. It covers the time span from 1990 to the present. Users can select specific parameters such as time points, geographical scope, age, and gender according to their needs through platforms like GBD Compare to query and download the corresponding incidence and prevalence data. The GBD database does not directly collect data on DALYs. Instead, it integrates multiple data sources and applies specific calculation methods to estimate DALYs. The details are as follows: Data sources: The GBD study utilizes a vast array of diverse data sources, including vital registration systems, verbal autopsies, censuses, household surveys, disease—specific registries, and health service contact data. These data sources cover numerous countries and regions across the globe. Calculation method: DALYs consists of two components: Years of Life Lost (YLL) due to premature death and Years Lost due to Disability (YLD) caused by disease—related disabilities. That is, DALYs = YLL + YLD. YLL is calculated by multiplying the number of deaths by specific cause—age—sex—location—year by the standard life expectancy at the age of death. YLD is obtained by multiplying the prevalence of sequelae by specific cause—age—sex—location—year by their respective disability weights. The disability weights, determined through expert reviews, are authoritative and range from 0 (perfect health) to 1 (death). Ethics approval and informed consent were not required for this study because of public accessibility to the data. All methods in this paper were performed following the relevant guidelines and regulations.

Our analysis made use of the GBD project's definition of PAD. Information regarding the three main clinical manifestations of PAD was collected from both males and females, across all age groups and regional groups covering 21 countries with similar epidemiological characteristics and in close geographical proximity. To differentiate in detail the burden of PAD as a function of age, patients were categorized in terms of age into subgroups of <70 years, 70–74 years, 75–79 years, and 80 + years. We also analyzed the disease burden of PAD patients aged over 70 and under 70. The socio-demographic index (SDI) is calculated by GBD 2021 for each country. This is a composite indicator comprising social and economic factors known to affect health outcomes in each place (8). The SDI is the normalized geometric mean of indices for the total fertility rate among people under the age of 25 years, the average years spent in education among people over 15 years of age, and the lagged per capita income, with 0 representing the combination of the highest fertility rate, the least time spent in education, and the lowest per capita income (9). Five quintiles are used to classify the SDI: low, low-middle, middle, high-middle, and high. Incidence, mortality, and DALYs are taken from GBD 2021, and each rate is shown per 100,000. The 2.5th and 97.5th values of the ordinal 1000 estimates calculated using the GBD are taken as the 95% uncertainty interval (UI) boundaries.

### Statistical analysis

Age-standardized incidence rate (ASIR), age-standardized mortality rate (ASMR), and age-standardized disability-adjusted life years (ASDALYs) were calculated per 100,000 in terms of sex, geographic location, and SDI. The estimated annual percentage change (EAPC) of these metrics was calculated to quantify the evolution of early onset PAD burden over time. The natural logarithm of the rates was fitted via linear regression, denoted as y = *α* + *β*x + *ε*, where y = ln (ASR), and x = calendar year. The EAPC is then 100 × [exp(*β*)−1], and the linear regression model provided the corresponding 95% confidence interval (CI).

Furthermore, research indicates that the Bayesian age-period-cohort (BAPC) framework can enhance the precision of statistical predictions with respect to alternative methods including generalized additive, joinpoint, and Poisson regression analyses (10). Therefore, this methodology, integrated with nested Laplace approximation techniques, was used to project PAD-related ASIR, ASMR and ASDALYs for the years 2022–2035. The R software package (version 4.2.3) and JD_GBDR (V2.36.2, Jingding Medical Technology Co., Ltd.) were used to draw the figures.

## Results

### Global trends

Globally, the ASIR of PAD has declined from 1990 to 2021 (EAPC, −0.4; 95% CI, −0.4 to −0.4) (Table 1); in 1990 the ASIR per 100 000 was 130.3 (95% UI, 112.8–149.4), dropping to 115.4 (95% UI, 100.0–132.7) in 2021. Both the ASMR (EAPC, −1.7; 95% CI, −1.8 to −1.5) and ASDALYs (EAPC, −1.4; 95% CI, −1.5 to −1.3) exhibited a similar downward trend. The ASMR reduced from 1.3 (95% UI, 1.2–1.4) in 1990 to 0.8 (95% UI, 0.7–0.9) in 2021. The ASDALYs declined from 26.6 (95% UI, 22.3–33.8) in 1990 to 18.6 (95% UI, 15.2–24.2) in 2021.

**Table 1:** Estimated number of global ASIR, ASMR, and ASDALYs for PAD and and its EAPCs in 1990–2021.

Characteristics	1990			2021			1990–2021 EAPCs		
	ASIR per 100 000 (95% UI)	ASMR per 100 000 (95% UI)	ASDALYs per 100 000 (95% UI)	ASIR per 100 000 (95% UI)	ASMR per 100 000 (95% UI)	ASDALYs per 100 000 (95% UI)	ASIR (95% CI)	ASMR (95% CI)	ASDALYs (95% CI)
Globalx	130.3 (112.8, 149.4)	1.3 (1.2, 1.4)	26.6 (22.3, 33.8)	115.4 (100.0, 132.7)	0.8 (0.7, 0.9)	18.6 (15.2, 24.2)	−0.4 (−0.4, −0.4)	−1.7 (−1.8, −1.5)	−1.4 (−1.5, −1.3)
Sex									
Male	96.9 (84.0, 111.4)	1.5 (1.4, 1.7)	29.5 (25.5, 34.6)	86.6 (75.4, 99.6)	1.0 (0.9, 1.1)	20.0 (17.3, 24.2)	−0.4 (−0.4, −0.4)	−1.7 (−1.8, −1.5)	−1.5 (−1.6, −1.4)
Female	158.4 (137.6, 182.1)	1.1 (1.0, 1.3)	24.0 (19.1, 32.3)	141.2 (122.0, 162.2)	0.7 (0.6, 0.8)	17.2 (13.0, 24.3)	−0.4 (−0.4, −0.4)	−1.7 (−1.9, −1.5)	−1.3 (−1.4, −1.2)
SDI									
High	192.0 (166.8, 220.1)	1.7 (1.5, 1.8)	34.6 (29.4, 43.2)	155.0 (135.9, 176.0)	1.3 (1.1, 1.4)	26.4 (22.4, 32.9)	−0.8 (−0.9, −0.7)	−1.0 (−1.3, −0.8)	−1.0 (−1.1, −0.8)
High-middle	125.4 (108.3, 144.1)	2.1 (1.9, 2.3)	39.9 (34.2, 47.1)	118.3 (102.2, 136.9)	1.1 (0.9, 1.1)	22.5 (19.1, 28.6)	−0.2 (−0.2, −0.2)	−2.7 (−2.9, −2.5)	−2.3 (−2.5, −2.1)
Middle	102.9 (89.0, 119.3)	0.4 (0.3, 0.4)	12.3 (8.8, 18.4)	103.6 (89.4, 120.1)	0.4 (0.3, 0.4)	11.5 (8.3, 16.9)	−0.0 (−0.1, 0.0)	−0.3 (−0.4, −0.2)	−0.3 (−0.4, −0.3)
Low-middle	85.8 (73.9, 99.9)	0.3 (0.2, 0.4)	9.1 (5.7, 14.0)	91.1 (78.5, 105.7)	0.4 (0.3, 0.5)	11.4 (8.0, 16.4)	0.2 (0.2, 0.2)	1.6 (1.5, 1.7)	0.7 (0.7, 0.8)
Low	74.3 (64.0, 86.3)	0.5 (0.2, 1.0)	12.7 (6.6, 21.5)	79.1 (68.0, 91.7)	0.7 (0.4, 1.4)	16.2 (9.3, 26.9)	0.2 (0.2, 0.2)	1.3 (1.1, 1.5)	0.8 (0.7, 0.9)
Region									
Central Asia	85.2 (72.9, 99.0)	0.2 (0.2, 0.3)	8.8 (6.1, 13.2)	91.6 (78.7, 106.6)	0.5 (0.4, 0.5)	12.5 (9.6, 16.9)	0.3 (0.3, 0.4)	2.7 (2.4, 3.0)	1.3 (1.2, 1.5)
Southeast Asia	118.1 (102.3, 137.3)	0.1 (0.1, 0.1)	9.2 (5.2, 16.4)	122.8 (105.9, 142.9)	0.1 (0.1, 0.2)	9.8 (5.7, 16.9)	0.2 (0.1, 0.2)	1.4 (1.4, 1.5)	0.2 (0.2, 0.3)
East Asia	109.8 (95.1, 127.4)	0.1 (0.1, 0.1)	9.0 (5.1, 16.0)	113.0 (98.0, 131.1)	0.1 (0.1, 0.1)	8.3 (4.9, 14.3)	0.0 (−0.1, 0.1)	0.8 (0.6, 1.0)	−0.4 (−0.4, −0.3)
Eastern Europe	100.6 (86.4, 116.1)	4.3 (3.7, 4.9)	76.6 (66.8, 88.5)	110.2 (95.0, 127.1)	2.8 (2.5, 3.1)	54.0 (48.4, 60.8)	0.4 (0.4, 0.4)	−2.0 (−2.3, −1.7)	−1.8 (−2.1, −1.5)
Central Europe	101.5 (87.3, 117.2)	2.7 (2.5, 2.9)	49.3 (44.3, 54.9)	100.1 (86.2, 115.6)	2.7 (2.4, 3.0)	48.7 (43.2, 55.4)	−0.1 (−0.1, −0.0)	−0.4 (−0.7, −0.1)	−0.5 (−0.8, −0.3)
Western Europe	207.6 (180.5, 236.6)	2.1 (1.9, 2.2)	41.4 (35.2, 51.5)	158.1 (136.6, 182.8)	1.4 (1.2, 1.6)	27.8 (23.1, 34.9)	−1.1 (−1.1, −1.0)	−1.0 (−1.4, −0.7)	−1.1 (−1.4, −0.9)
Southern Latin America	152.0 (131.8, 175.7)	0.6 (0.5, 0.6)	16.4 (12.3, 24.0)	134.4 (116.2, 155.8)	0.5 (0.4, 0.5)	13.6 (10.3, 19.6)	−0.5 (−0.5, −0.4)	0.0 (−0.5, 0.5)	−0.4 (−0.7, −0.2)
Oceania	102.9 (89.1, 120.1)	0.1 (0.1, 0.2)	8.6 (4.9, 15.0)	111.0 (96.1, 129.4)	0.1 (0.1, 0.2)	9.7 (5.5, 16.8)	0.3 (0.2, 0.3)	0.5 (0.1, 0.9)	0.4 (0.2, 0.5)
High-income Asia Pacific	161.6 (139.7, 184.4)	0.4 (0.3, 0.4)	13.2 (9.0, 20.5)	109.3 (94.0, 125.7)	0.3 (0.3, 0.4)	9.4 (6.8, 13.7)	−1.5 (−1.5, −1.4)	0.0 (−0.4, 0.4)	−1.1 (−1.3, −1.0)
Australasia	145.8 (125.5, 167.9)	3.3 (2.9, 3.6)	51.1 (45.7, 57.5)	105.6 (90.7, 121.7)	1.6 (1.3, 1.8)	24.2 (20.6, 28.7)	−1.2 (−1.3, −1.1)	−2.6 (−2.8, −2.4)	−2.6 (−2.8, −2.4)
High-income North America	229.8 (199.7, 265.0)	1.8 (1.6, 1.9)	37.1 (31.7, 46.0)	207.2 (184.8, 231.5)	1.7 (1.5, 1.8)	35.6 (30.5, 43.4)	−0.3 (−0.4, −0.2)	−0.7 (−1.1, −0.4)	−0.6 (−0.8, −0.4)
Caribbean	86.7 (74.5, 100.3)	1.7 (1.5, 1.8)	29.9 (26.8, 34.3)	90.4 (77.3, 104.9)	1.9 (1.7, 2.1)	34.8 (30.5, 40.4)	0.1 (0.1, 0.1)	0.5 (0.4, 0.6)	0.6 (0.4, 0.7)
Andean Latin America	66.8 (57.4, 77.6)	0.1 (0.1, 0.1)	4.9 (2.9, 8.6)	71.8 (61.7, 83.6)	0.1 (0.1, 0.2)	5.7 (3.9, 9.1)	0.3 (0.2, 0.3)	2.6 (2.2, 3.0)	0.6 (0.5, 0.8)
Central Sub-Saharan Africa	77.5 (66.4, 89.7)	1.2 (0.6, 2.3)	25.7 (13.9, 43.8)	79.8 (68.9, 93.2)	1.8 (1.0, 3.1)	34.8 (20.6, 57.8)	0.1 (0.0, 0.1)	1.3 (0.9, 1.6)	1.0 (0.8, 1.3)
North Africa and Middle East	83.0 (71.2, 96.5)	0.2 (0.1, 0.3)	8.1 (5.0, 12.6)	97.3 (83.5, 112.9)	0.3 (0.2, 0.3)	9.4 (6.8, 13.9)	0.5 (0.5, 0.6)	2.1 (1.7, 2.5)	0.8 (0.6, 0.9)
Central Latin America	96.0 (82.5, 111.1)	0.8 (0.7, 0.8)	16.4 (13.5, 21.5)	91.7 (78.4, 106.1)	0.4 (0.4, 0.5)	11.0 (8.4, 15.6)	−0.2 (−0.2, −0.1)	−2.3 (−2.7, −2.0)	−1.6 (−1.8, −1.3)
Tropical Latin America	101.2 (86.9, 117.1)	1.5 (1.3, 1.6)	30.3 (26.8, 35.5)	88.5 (76.2, 102.8)	1.3 (1.1, 1.4)	26.7 (23.4, 31.0)	−0.5 (−0.6, −0.5)	−0.6 (−0.9, −0.4)	−0.7 (−0.9, −0.5)
South Asia	79.9 (68.8, 93.1)	0.1 (0.1, 0.2)	6.9 (3.9, 11.6)	84.6 (72.9, 98.2)	0.3 (0.2, 0.4)	8.6 (5.6, 13.4)	0.1 (0.1, 0.2)	2.4 (2.2, 2.6)	0.8 (0.7, 0.8)
Southern Sub-Saharan Africa	107.3 (92.5, 123.0)	1.3 (1.0, 1.6)	32.2 (25.0, 39.4)	98.6 (85.0, 114.2)	2.0 (1.8, 2.3)	44.7 (38.7, 51.7)	−0.4 (−0.4, −0.3)	1.6 (1.3, 1.8)	1.3 (1.0, 1.5)
Eastern Sub-Saharan Africa	71.0 (61.1, 82.5)	0.8 (0.4, 1.6)	17.4 (9.2, 31.0)	74.2 (63.8, 86.1)	1.2 (0.7, 2.2)	23.2 (14.1, 40.1)	0.1 (0.1, 0.2)	1.3 (1.1, 1.4)	1.0 (0.9, 1.1)
Western Sub-Saharan Africa	69.3 (59.5, 80.7)	1.0 (0.5, 1.9)	18.8 (10.6, 34.0)	76.0 (65.3, 88.2)	1.4 (0.8, 2.8)	25.7 (15.5, 47.3)	0.3 (0.3, 0.3)	1.2 (1.2, 1.2)	1.0 (0.9, 1.0)

ASIR, age-standardized incidence rate; ASMR, age-standardized mortality rate; ASDALYs, age-standardized disability-adjusted life-years; PAD, peripheral arterial disease; EAPC, estimated annual percentage change; SDI, socio-demographic index; UI, uncertainty interval; CI, Confidence interval.

**Figure 1:**
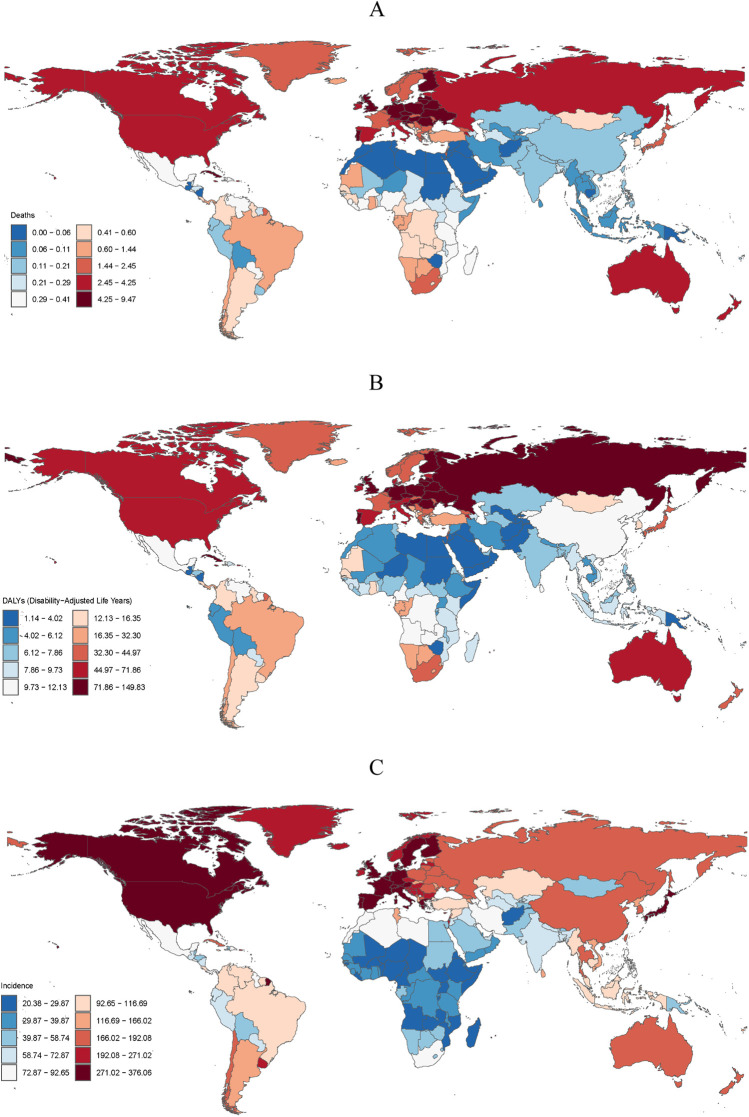
The distribution of EAPCs in 204 countries and regions. EAPCs of ASMR **(A)**, ASDALYs **(B)** and ASIIR **(C)** of peripheral arterial disease. EAPC, estimated annual percentage change; ASMR, age-standardized mortality rate; ASDALYs, age-standardized disability-adjusted life-years; ASIR, age-standardized incidence rate. Each color corresponds to a specific rage of EAPC values, with the EAPC increasing as the color changes from blue to brown. World map created using the rnaturalearth R package, https://github.com/ropensci/rnaturalearth.

The EAPC of ASMR map of 204 countries and regions shows that the three countries with the highest EAPC in 2021 were Georgia (16.7), Afghanistan (10.3) and Sudan (9.7) (Figure 1A). The corresponding countries in terms of the EAPC of ASDALYs (Figure 1B) and ASIR (Figure 1C) were Georgia (5.5), Bahrain (3.4) and Mongolia (3.1), and Lebanon (1), Afghanistan (0.9) and Egypt (0.8), respectively.

### Global trends by gender

Overall, the ASIR, ASMR and ASDALYs for both males and females declined from 1990 to 2021 (Table 1). The EAPCs of ASIR were the same for males and females (EAPC, −0.4; 95% CI, −0.4 to −0.4), and the EAPCs of ASMR were similar for males (EAPC, −1.7; 95% CI, −1.8 to −1.5) and females (EAPC, −1.7; 95% CI, −1.9 to −1.5). Finally, the EAPC of ASDALYs in males was −1.5 (95% CI, −1.6 to −1.4) and that in females was −1.3 (95% CI, −1.4 to −1.2). In comparison, the ASIR (96.9; 95% UI, 84.0–111.4), ASMR (1.5; 95% UI, 1.4–1.7) and ASDALYs (29.5; 95% UI, 25.5–34.6) of males in 1990 were higher than those in 2021 (86.6; 95% UI, 75.4–99.6), (1.0; 95% UI, 0.9–1.1) and (20.0; 95% UI, 17.3–24.2), respectively. A similar trend was observed in females: ASIR (158.4; 95% UI, 137.6–182.1), ASMR (1.1; 95% UI, 1.0–1.3), and ASDALYs (24.0; 95% UI, 19.1–32.3) in 1990; (141.2; 95% UI, 122.0–162.2), (0.7; 95% UI, 0.6–0.8), and (17.2; 95% UI, 13.0–24.3) in 2021, respectively.

### Global trends by SDI and GBD 21 regions

The SDI in terms of ASIR, ASMR, and ASDALYs has evolved over the past three decades (Table 1). Among these factors, the ASIR (EAPC, 0.2; 95% CI, 0.2–0.2), ASMR (EAPC of low SDI, 1.3; 95% CI, 1.1–1.5; EAPC of middle-low SDI, 1.6; 95% CI, 1.5–1.7) and ASDALYs (EAPC of low SDI, 0.8; 95% CI, 0.7–0.9; EAPC of middle-low SDI, 0.7; 95% CI, 0.7–0.9) in low and middle-low SDI areas all exhibited an upward trend. In contrast, the incidence, mortality and DALYs in high, high-middle and middle SDI areas declined. In both 1990 and 2021, it was observed that the SDI was positively correlated with the incidence rate, mortality rate and DALYs, excluding low SDI areas.

Analysis of the trends of ASIR, ASMR, and ASDALYs in 21 regions globally revealed a correlation between socio-economic development and the rates of ASIR, ASMR, and ASDALYs. For instance, North America had the highest ASIR (229.8; 95% UI, 199.7–265.0; 207.2; 95% UI, 184.8–231.5.0) in both 1990 and 2021. Fortunately, over the past 30 years, these overall metrics in economically and socially developed regions have declined, including the high-income Asia Pacific region (EAPC of ASIR, −1.5; 95% CI, −1.5 to −1.4), Australasia (EAPC of ASIR, −1.2; 95% CI, −1.3 to −1.1), and Western Europe (EAPC of ASIR, −1.1; 95% CI, −1.1 to −1.0) etc.

### Global trends by age group

**Figure 2:**
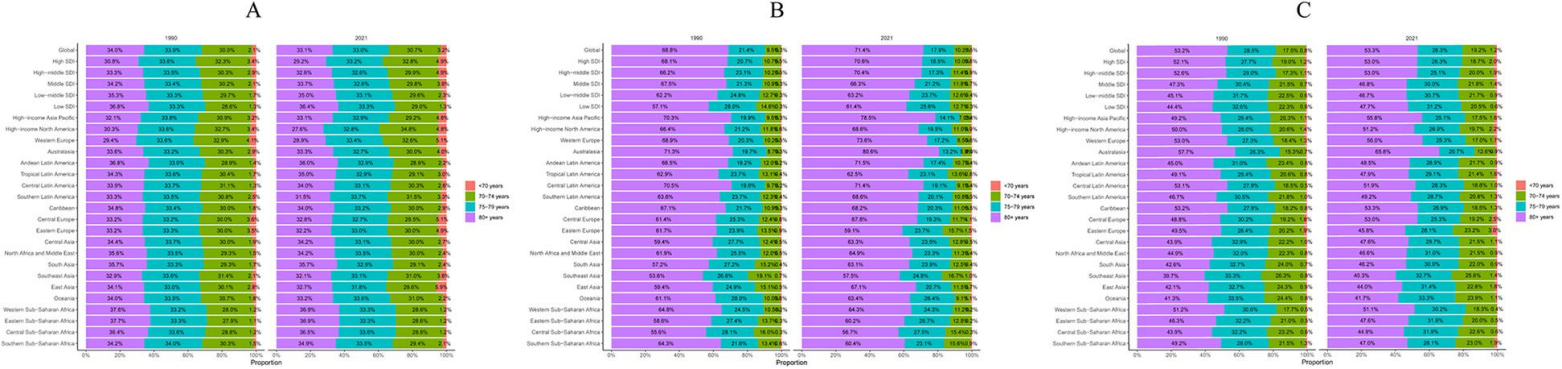
The proportion of incidence **(A)**, mortality **(B)** and DALYs **(C)** in different age groups in global and GBD 21 regions in 1990 and 2021. DALYs, disability-adjusted life-years; GBD, global burden of disease.

The incidence, mortality rate and DALYs of PAD in the global population aged under 70 years old were all less than 10% across the time period studied (Figure 2). No region-related differences were found in terms of age across the 21 GBD regions. Among the three groups of people aged 70–74, 75–79 and 80+, the incidence rates were almost equal (Figure 2A), with people aged 80+ accounting for over 50% of PAD deaths (Figure 2B). The DALYs were positively correlated with the age of the PAD population (Figure 2C).

**Figure 3:**
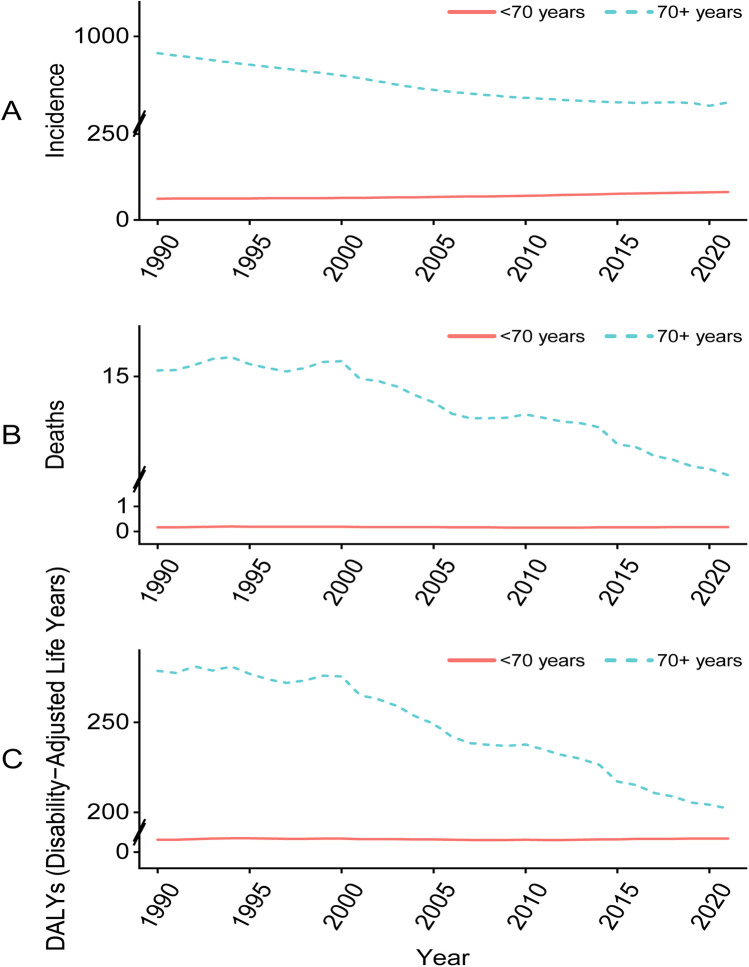
Trend analysis of the incidence **(A)**, mortality **(B)**, and DALYs **(C)** of global peripheral arterial disease for time and age. In the figure, a break point is applied to the vertical axis (A, *Y*-axis: 0 to 275, 751 to 1100; B, *Y*-axis: −0.5 to 2, 11 to 17; C, *Y*-axis: −9 to 10, 190 to 290) to optimize the visual presentation. DALYs, disability-adjusted life-years.

Given the apparent significant epidemiological differences between PAD patients aged <70 years and those aged ≥70 years, an analysis was conducted of the temporal trends of the incidence, mortality, and DALYs across these two groups (Figure 3). All of these metrics exhibited an upward trend for under 70s, while the opposite was true for people over 70. In other words, PAD was increasingly affecting younger people.

### Risk factor trends analysis by SDI

**Figure 4:**
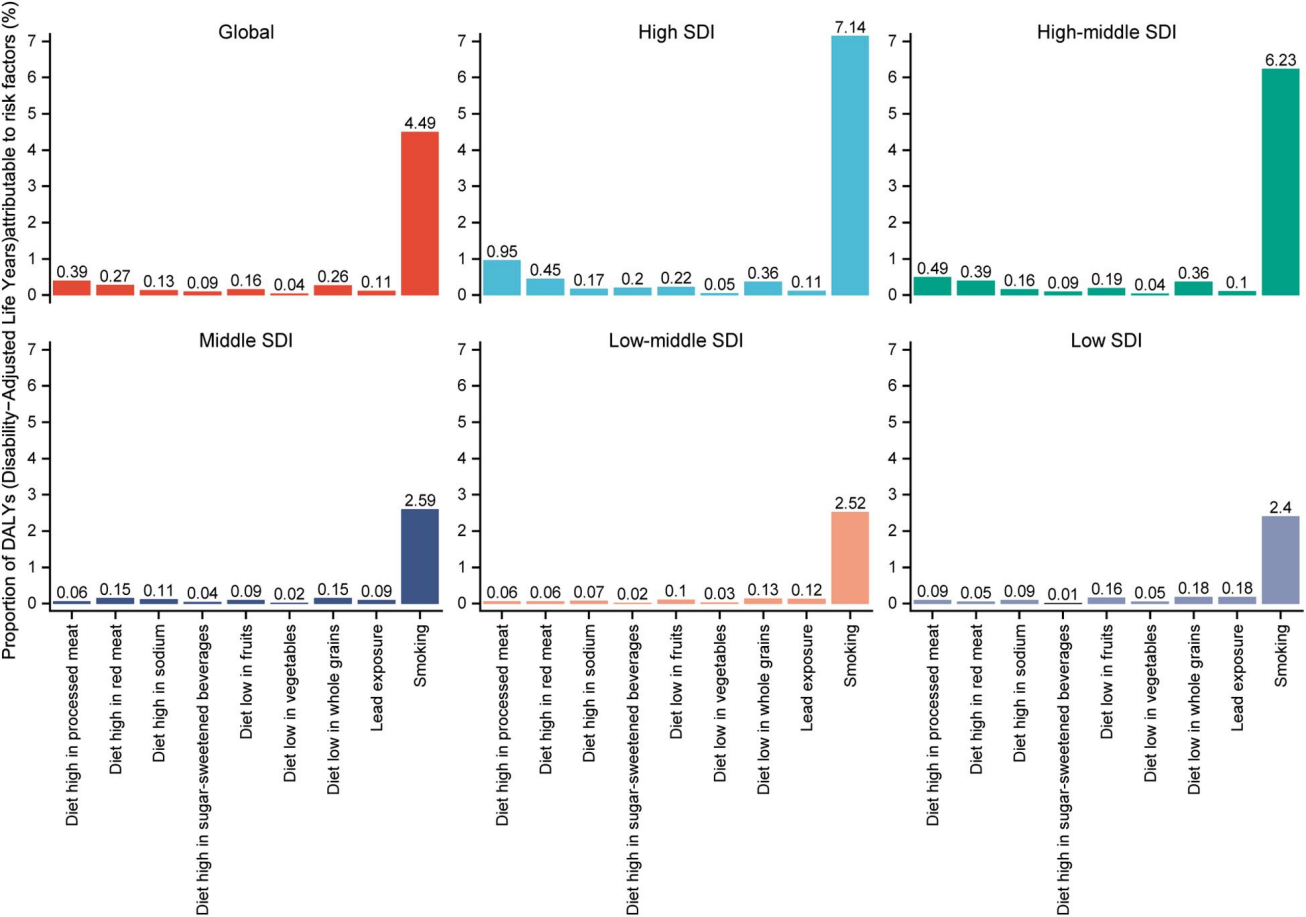
Analysis of risk factors for DALYs globally and in diferrent SDI regions. DALYs, DALYs, disability-adjusted life-years; SDI, socio-demographic index.

Smoking was the greatest risk factor for DALYs of PAD, both across the globe and across all levels of SDI (Figure 4). The next most significant risk factors globally were diets consisting of high proportions of processed meat and red meat, respectively. Similar results were observed in high and high-middle SDI regions, while in medium SDI regions, the second and third most significant risk factors were diets low in whole grains and high in red meat. Finally, in low-middle and low SDI areas, these factors were a diet low in whole grains, and lead exposure.

**Figure 5:**
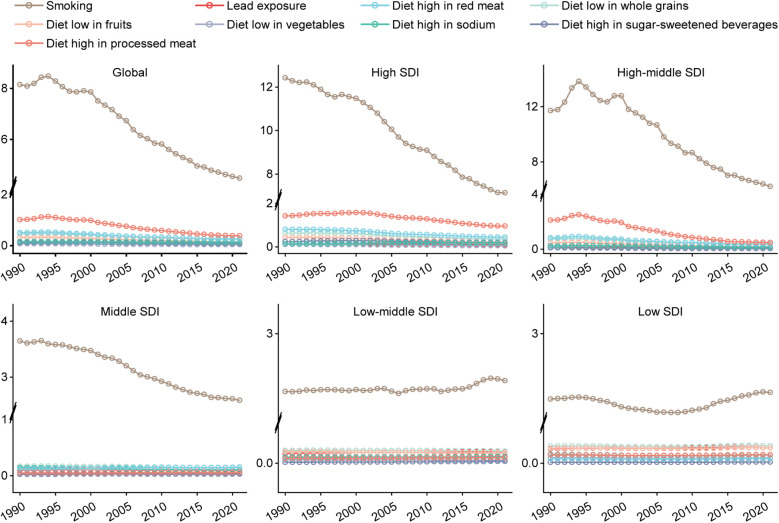
Analysis of risk factor trends in global and different SDI regions from 1990 to 2021. In the figure, a break point is applied to the vertical axis (Global, *Y*-axis: −0.3 to 2.3, 4.2–8.9; High SDI, *Y*-axis: −0.3 to 2.1, 6.9–13; Middle SDI, *Y*-axis: −0.3 to 4.1, 6–15; Low-Middle SDI, *Y*-axis: −0.3 to 0.4, 2.1–3.3; Low SDI, *Y*-axis: −0.3 to 0.4, 2.1–3.3) to optimize the visual presentation. SDI, socio-demographic index.

The temporal trends of risk factors between 1990 and 2021 were also analyzed. Smoking remains the biggest risk factor globally, and for high, high-middle and middle SDI areas, although it has shown a downward trend (Figure 5). However, in the low-middle and low SDI areas, the trend of this risk factor fluctuated but generally showed an upward trend.

### Prediction of the ASIR, ASMR and ASDALYs of PAD from 2022 to 2035

**Figure 6:**
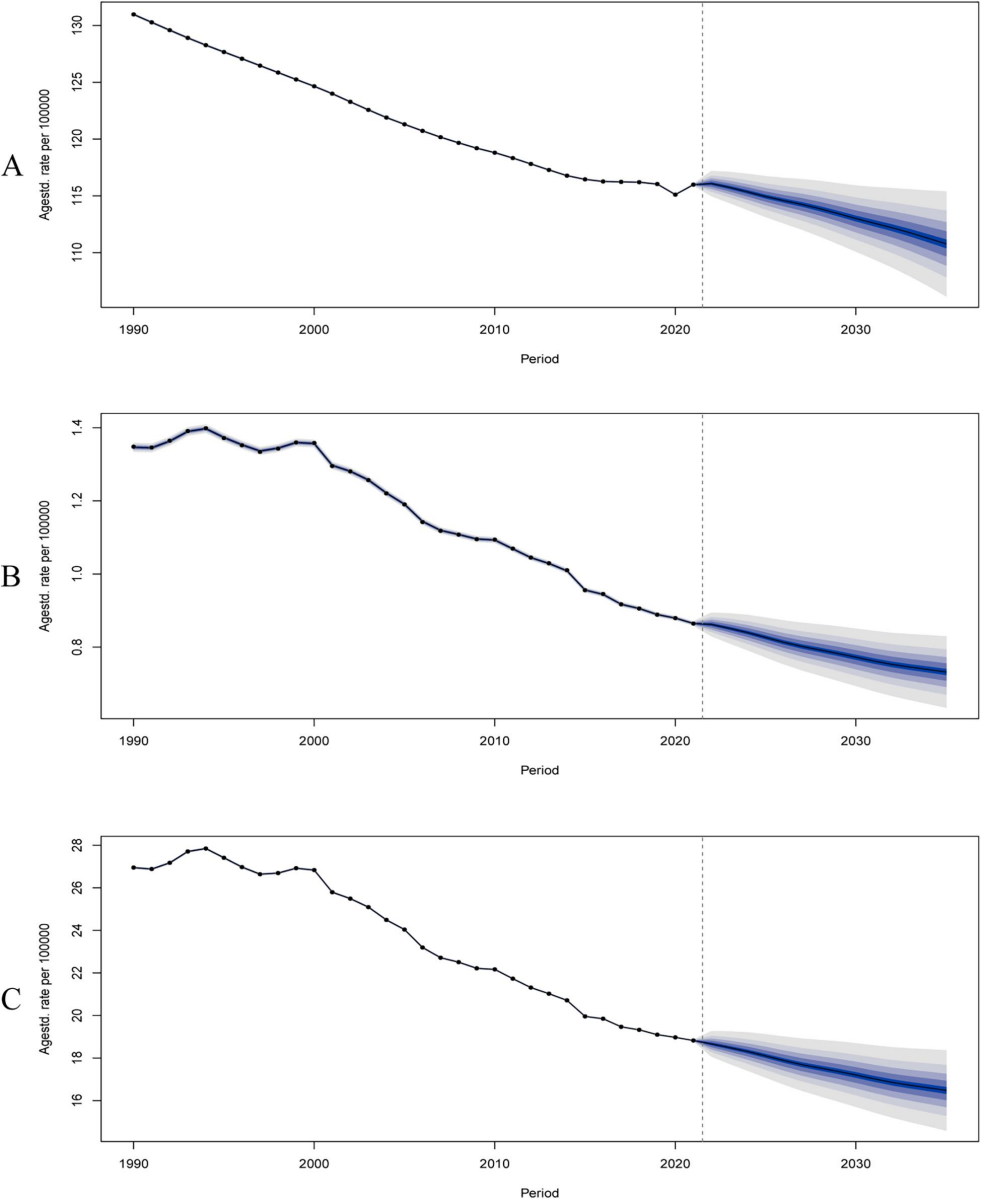
Predict the global ASIR **(A)**, ASMR **(B)** and ASDALY **(C)** trends from 2022 to 2035. ASIR, age-standardized incidence rate; ASMR, age-standardized mortality rate; ASDALYs, age-standardized disability-adjusted life-years. The shaded areas after 2022 represent 95% confidence intervals.

The ASIR, ASMR and ASDALYs of PAD are predicted to show a downward trend (Figure 6). Specifically, the ASIR is expected to fall from 115.2 (95% CI, 114.8–116.9) in 2022 to 110.5 (95% CI, 107.0–114.1) in 2035; ASMR from 0.8 (95% CI, 0.8–0.9) in 2022 to 0.7 (95% CI, 0.6–0.8) in 2035; and ASDALYs from 18.5 (95% CI, 17.9–19.4) in 2022 to 16.5 (95% CI, 14.2–18.7) in 2035. In the low-middle and low SDI regions, both the incidence rate, mortality rate and DALYs showed an upward trend, while in the high, high-middle and middle SDI regions, they showed a downward trend (Supplementary Figure S1–S3).

## Discussion

This retrospective analysis of recent trends in PAD around the world has revealed an overall decrease in ASIR, ASMR and ASDALYs between 1990 and 2021. Projections of these metrics worldwide up to 2035 have also been made, with this trend expected to continue. Therefore, to some extent the burden of PAD can be reduced further under the guidance of existing guidelines and clinical practice (11, 12). Despite the overall global downward trend of PAD, the opposite trend was observed in regions with different levels of SDI. Table 1 shows that the ASIR, ASMR and ASDALYs of PAD in high, high-middle and middle SDI areas followed the global downward trend, as reported in previous studies (13–18), but low-middle and low SDI areas showed an upward trend. These research findings not only point out the direction for future research, which involves focusing on exploring the driving factors behind the rise of PAD in regions with low-middle and low SDI and formulating targeted prevention and control programs, but also assist patients and the general public in different SDI regions in identifying key areas of health concern. Moreover, they provide a reference for more precisely reducing the overall disease burden of PAD.

One explanation for the upward trend of the disease burden of PAD in low-middle and low SDI regions is globalization: the eating habits and lifestyles of low- and middle-income countries have changed tremendously in recent decades (19–22). Globalization commonly leads to the gradual replacement of plant-based diets by unhealthy diets, including higher proportions of animal foods and foods containing added sugar, as confirmed in Figure 4 of our risk factor analysis. Other factors contributing to this trend in lower SDI areas are socio-economic: lower incomes, lower education levels, and less social support are all associated with a higher PAD-related disease burden rate (23, 24). Furthermore, populations in areas with lower SDI are faced with an increased risk of cardiovascular diseases owing to factors including smoking (25), hypertension (26) and diabetes (27). Prevention, detection and treatment of PAD, can be achieved with early screening and management of these major risk factors, thereby reducing the disease burden (28). Targeted public health strategies should be adopted in low SDI areas, including low-cost PAD screening of PAD, management of risk factors, and standardized treatment (29, 30).

Our study revealed that PAD shows an upward trend among individuals under the age of 70. The incidence, mortality and DALYs of people younger than 70 years old and ≥70 years old were analyzed, demonstrating an overall decline in the PAD population aged ≥70 years old and an overall increase in people younger than 70 years old. Previous studies on atherosclerosis made a similar discovery (31), revealing that both atherosclerosis incidence and mortality in people ≥55 years old have decreased over the past 30 years. The importance of prevention and early treatment of high-risk populations was highlighted, but the incidence of atherosclerosis was also found to be rising rapidly in young people aged between 20 and 54. This trend was correlated with an increase in risk factors such as obesity, diabetes, and hypertension (32) in young people that deserves immediate attention from policymakers.

The analysis of PAD risk factors revealed that smoking was the primary risk factor across regions with different SDI levels, as demonstrated in previous clinical literature (33, 34). Nicotine and other harmful substances found in tobacco can damage the endothelial cells in the blood vessels, leading to inflammation and fibrosis of the blood vessel walls, in turn giving rise to atherosclerotic plaques (37). These plaques can then enlarge gradually and block blood vessels, reducing the blood supply to the lower limbs and causing arteriosclerosis and occlusion of the lower limbs. Long-term smoking can also increase the risk of other cardiovascular diseases (35). Quitting smoking is an important measure for disease prevention and treatment. Clinical studies have found that the majority of patients who relapsed after surgery did not quit smoking (36). Therefore, patients with smoking habits should undergo smoking cessation measures actively, and vascular examinations of lower limbs should be performed regularly for the early detection and treatment of lesions in the early stage. According to Figure 4, it can be observed that the proportion of dietary risk factors and lead exposure varies in different SDI regions. In high and high-middle SDI regions, diets high in processed meat and red meat account for a larger proportion, while in low and low-middle SDI regions, diets low in whole grains and lead exposure are more prevalent. Therefore, when formulating public health policies, different regions need to target their policies based on the characteristics of their own risk factors.

The results of this study are limited by several factors. First, the information considered for our analysis is limited to identifying and comparing the global trends in ASIR, ASMR and ASDALYs of PAD between 1990 and 2021, and causal statements cannot be made about these data. Secondly, the accuracy of the distribution of the root causes of death may vary within and between countries. In the course of chronic diseases such as PAD, the presence of other diseases may complicate the accuracy of death certificates. Previous study has estimated that in 2012 only 38% of global deaths were registered, with the civil registration and demographic dynamics statistics system performing best in Europe, the Americas and Australia (37). Third, changes in coding practices and systems across time and in different regions may also affect the robustness of the data. Fourth, GBD tools do not stratify PAD depending on the symptoms of the affected individual, and it is therefore difficult to judge the consistency of the diagnostic criteria used to diagnose PAD across different countries. Especially in low-SDI countries, there are big limitations on data quality. Finally, as with any observational study, a large number of unmeasured confounders for factors may be present that are beyond the scope of this analysis.

## Conclusions

The incidence, mortality, and DALYs of PAD have shown a downward trend in recent decades, and this is expected to continue into the future. However, there has been a significant upward trend in the disease burden in regions with low and low-middle SDI. Therefore, more targeted public health strategies are required for areas with lower SDI and for those aged less than 70 years old, including strictly quitting smoking.

## Data Availability

The datasets presented in this study can be found in online repositories. The names of the repository/repositories and accession number(s) can be found below: the datasets analyzed during the current study are available on the website of the Global Health Data Exchange (https://vizhub.healthdata.org/gbd-results/).
