# Supplementary figures and images for: Global and national burden of peripheral arterial disease from 1990 to 2021: a systematic analysis of global burden of disease study 2021
Source: Front Cardiovasc Med. 2025 Oct 27;12:1603810. doi: 10.3389/fcvm.2025.1603810 (PMC12598569; doi:10.3389/fcvm.2025.1603810)

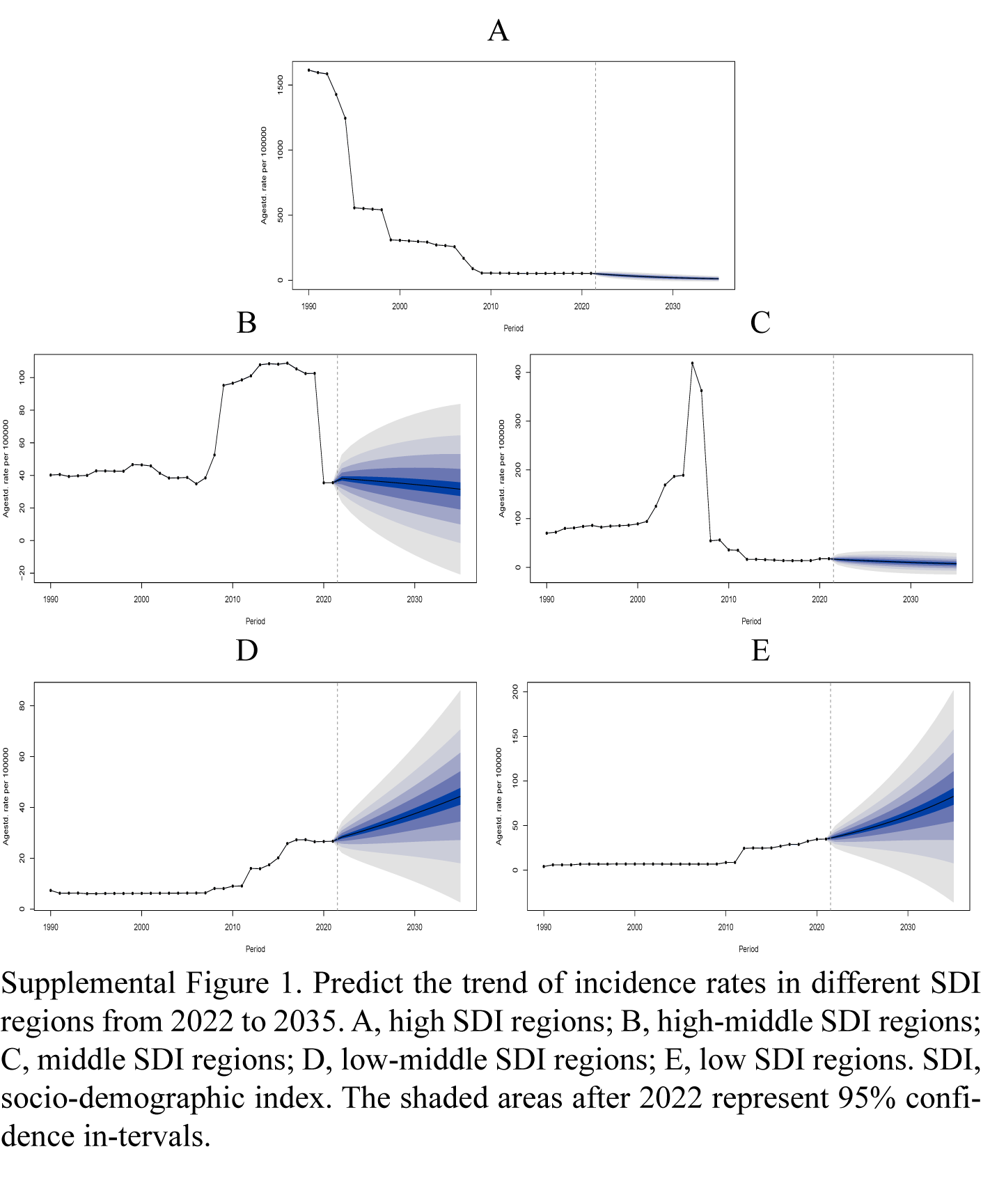

Supplement: Supplementary file 1 [file Image1.tif]

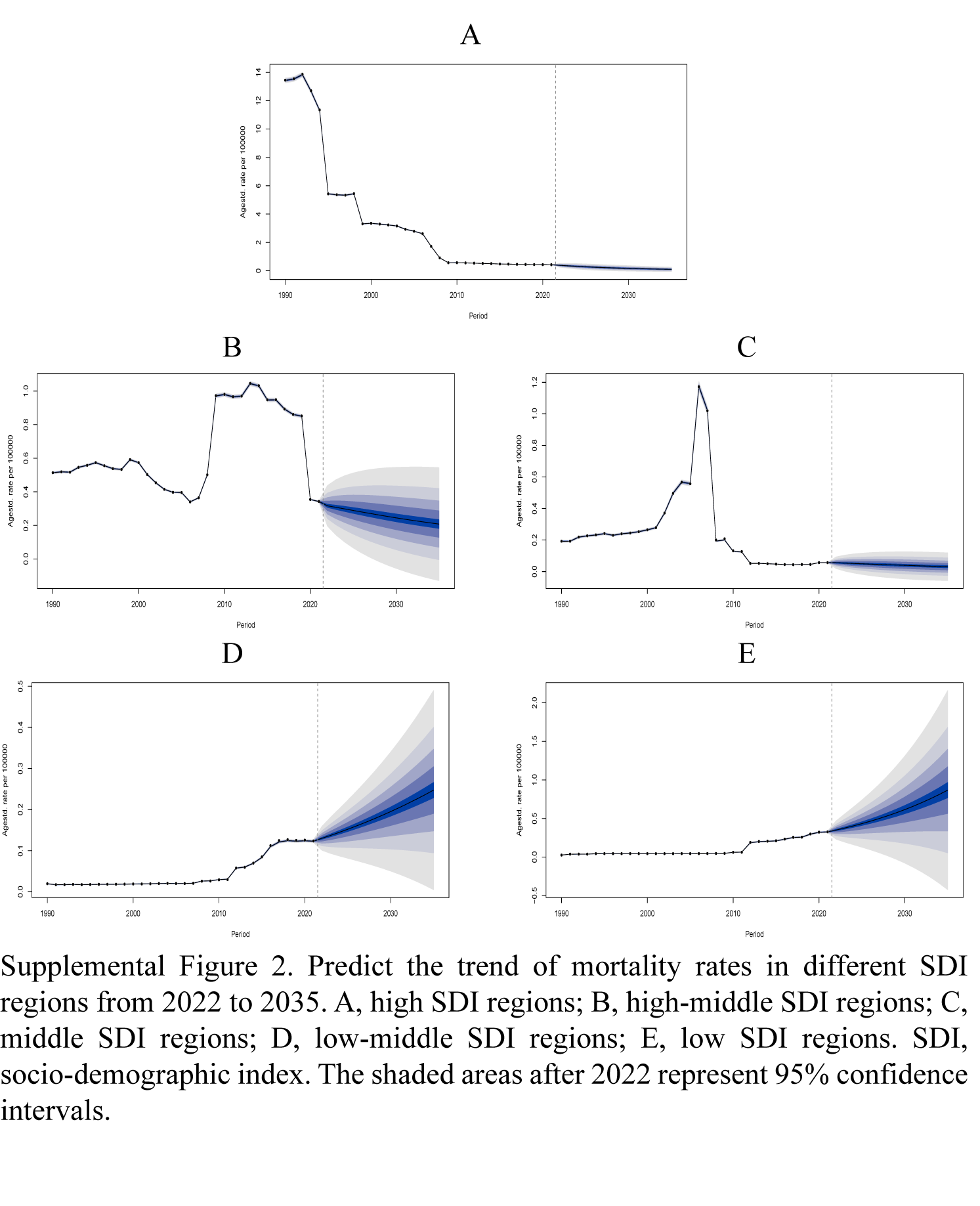

Supplement: Supplementary file 2 [file Image2.tif]

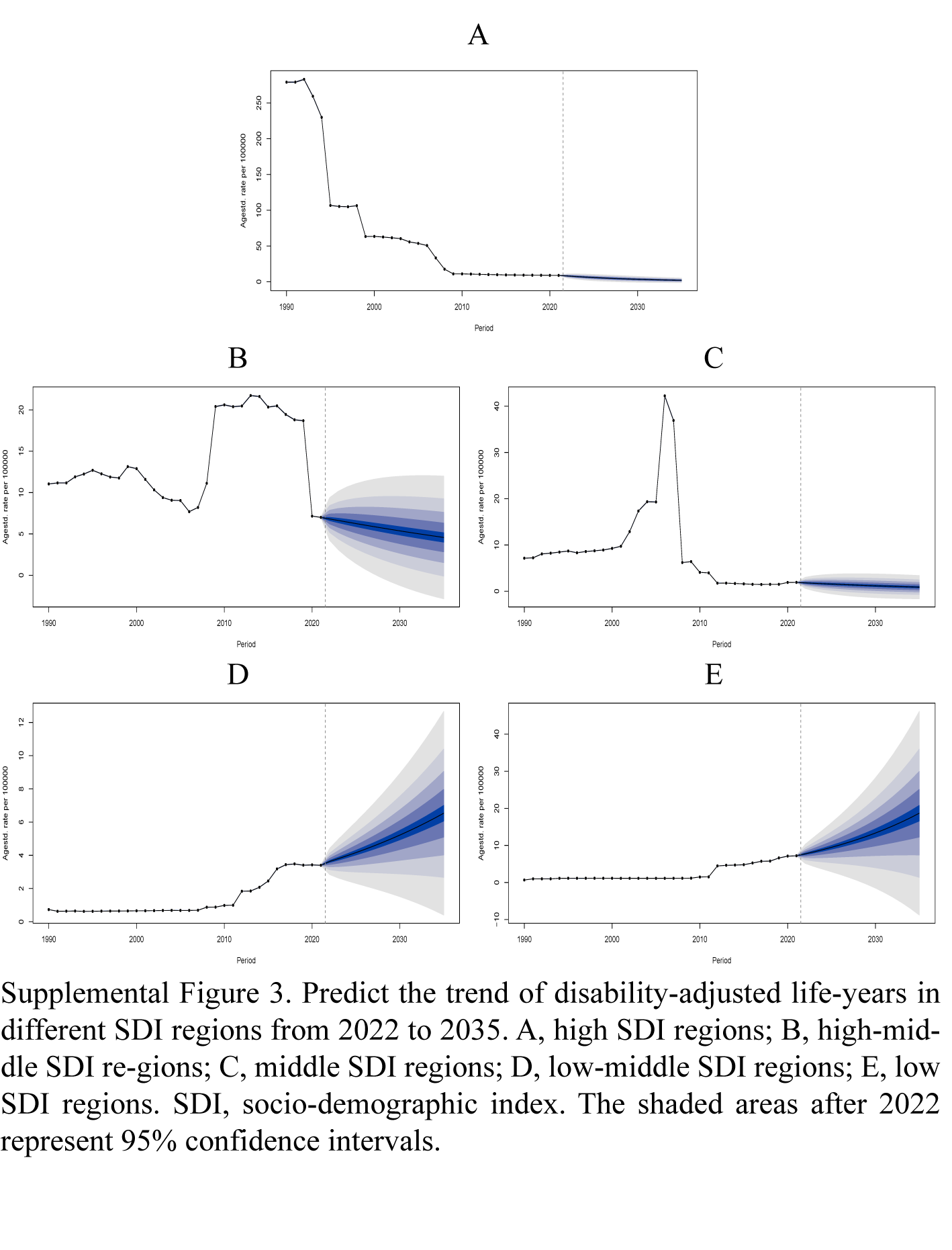

Supplement: Supplementary file 3 [file Image3.tif]
